# Galectin 9 Levels as a Potential Predictor of Intact HIV Reservoir Decay

**DOI:** 10.1093/infdis/jiae426

**Published:** 2025-02-04

**Authors:** Sergio Serrano-Villar, Akshay Gala, Peter Bacchetti, Rebecca Hoh, Clara di Germanio, Lillian B. Cohn, Timothy J. Henrich, Peter W. Hunt, Gregory M. Laird, Satish K. Pillai, Steven G. Deeks, Michael J. Peluso

**Affiliations:** 1 Division of HIV, Infectious Diseases, and Global Medicine, Department of Medicine, Zuckerberg San Francisco General Hospital, and University of California San Francisco; 2 Department of Infectious Diseases, Hospital Universitario Ramón y Cajal, and IRICYS; 3 CIBER de Enfermedades Infecciosas, Instituto de Salud Carlos III, Madrid, Spain; 4 Vitalant Research Institute and University of California San Francisco; 5 Department of Epidemiology and Biostatistics, University of California San Francisco; 6 Vaccine and Infectious Disease Division, Fred Hutchinson Cancer Center, Seattle, Washington; 7 Division of Experimental Medicine, Department of Medicine, Zuckerberg San Francisco General Hospital, and University of California San Francisco; 8 Accelevir Diagnostics, Baltimore, Maryland

**Keywords:** cytokines, galectin 9, HIV persistence, HIV reservoir, inflammation

## Abstract

**Background.:**

During antiretroviral therapy (ART), the HIV reservoir shows variability, with cells carrying intact genomes decaying faster than those with defective genomes, particularly in the first years. The host factors influencing this decay remain unclear.

**Methods.:**

Observational study of 74 PWH on ART, 70 (94.6%) of whom were male. Intact proviruses were measured using the intact proviral DNA assay, and 32 inflammatory cytokines were quantified using Luminex immunoassay. Linear spline models assessed the impact of baseline cytokine levels and their trajectories on intact HIV kinetics over seven years.

**Results.:**

Baseline Gal-9 was the strongest predictor, with lower levels predicting faster decay. A 10-fold decrease in baseline Gal-9 correlated with a 45% (95% CI, 14%–84%) greater annual decay of intact HIV genomes. Higher baseline interferon-inducible T-cell α chemoattractant (ITAC), interleukin 17 (IL-17), and macrophage inflammatory protein 1α (MIP-1α) levels also predicted faster decay. Longitudinal increases in MIP-3α and decreases in IL-6 were linked to a 9.5% and 10% faster decay, respectively.

**Conclusions.:**

The association between lower baseline Gal-9 and faster intact HIV decay suggests targeting Gal-9 could enhance reservoir reduction. The involvement of MIP-3α and IL-6 highlights a broader cytokine regulatory network, suggesting potential multi-targeted interventions.

Although antiretroviral therapy (ART) substantially reduces morbidity and mortality, complete eradication remains unattainable because of the HIV reservoir. These latently infected cells persist despite suppressive ART and result in viral rebound upon cessation of ART [1, 2]. Determining the predictors and dynamics of changes in the HIV reservoir is a major priority for the field [3].

The quantitative viral outgrowth assay—the gold standard method to quantify replication-competent HIV—is resource intensive and requires large volumes of blood, which limits its throughput and feasibility for use in large clinical trials [4]. Cell-associated total HIV DNA measurements, while easier to perform, overestimate the size of the reservoir and cannot distinguish replication competent (ie, clinically relevant) from nonintact genomes. In contrast, the recent introduction of the intact proviral DNA assay (IPDA) has allowed for a higher throughput and more precise estimation of the HIV genomes most likely to contribute to viral rebound [5]. By using droplet digital polymerase chain reaction to simultaneously examine the packaging signal and *env* regions, the IPDA separately quantifies intact and defective proviruses. Despite limitations [6, 7], this approach allows the IPDA to efficiently provide a more accurate estimate of the replication-competent reservoir [8]. It accomplishes this by differentiating between intact sequences and defective ones that are unlikely to be clinically significant.

Identifying individuals likely to experience significant HIV reservoir decay during ART would aid cure efforts. Predicting reservoir decay trajectories could help select candidates for cure interventions or assess an intervention’s additive effect. Inflammatory cytokine levels, which influence reservoir persistence, are potential predictors of intact HIV reservoir dynamics. Here, we aimed to identify cytokines as reliable predictors for changes in the intact HIV reservoir. Baseline galectin 9 (Gal-9) levels emerged as the most predictive marker of intact HIV kinetics over 7 years. Lower baseline Gal-9 levels predicted greater decay of intact HIV genomes over 7 years of suppressive ART, while decreases in macrophage inflammatory protein 3α (MIP-3α) and interleukin 6 (IL-6) over time correlated with concurrent decreases of intact HIV genomes.

## METHODS

### Study Participants and Samples

Participants included those enrolled in the University of California San Francisco SCOPE cohort from 2001 to 2017. SCOPE is a comprehensive longitudinal cohort of people with HIV with detailed clinical, virologic, and immunologic data. We identified individuals undergoing ART for at least 1 year with plasma HIV RNA levels below the quantification limit at the baseline visit, who maintained viral suppression for at least 2 years, allowing for isolated “blips” of HIV RNA above the quantification limit but <200 copies/mL. We prioritized participants with the longest viral suppression duration and >2 stored samples of peripheral blood mononuclear cells and plasma spanning this period. Basic HIV parameters, including plasma HIV RNA and CD4+ T-lymphocyte counts, were measured with clinical assays at specimen collection. Sex and gender were self-reported by participants.

### IPDA Measurements

An in-depth description of the IPDA rationale and procedure is available from Bruner et al [5]. For this analysis, we used IPDA measurements performed by Accelevir Diagnostics under company standard operating procedures as part of a previous effort to characterize intact proviral decay [6]. Briefly, cryopreserved peripheral blood mononuclear cells were viably thawed, and total CD4+ T cells were obtained via immunomagnetic selection (EasySep Human CD4+ T-Cell Enrichment Kit; Stemcell Technologies), with cell count, viability, and purity assessed by flow cytometry before and after selection. An average of 2.5 million untouched CD4+ T cells were recovered from each sample. Genomic DNA was isolated by the QIAamp DNA Mini Kit (Qiagen). DNA concentrations were determined by fluorometry (Qubit dsDNA BR Assay Kit; Thermo Fisher Scientific), and DNA quality was determined by ultraviolet-visible spectrophotometry (QIAxpert; Qiagen). Genomic DNA was then analyzed by IPDA as previously described [5, 6].

### Cytokine Measurements

Luminex assays were conducted at the Vitalant Research Institute. We chose 32 targets from Human Luminex Discovery assay panels based on a review of literature on cytokines pertinent to the HIV reservoir. Due to bead region incompatibilities, the cytokines were divided into 26- and 6-plex assays. Five batches per panel were ordered from R&D Systems to process 200 plasma samples in duplicate. Luminex kits were utilized following the manufacturer’s recommendations. Plasma samples were centrifuged at 16 000*g* for 4 minutes. Samples and standards were incubated with an antibody-linked magnetic bead cocktail for 2 hours at room temperature on a horizontal orbital shaker set at 800 rpm. Wells were washed 3 times with buffer, followed by a 1-hour incubation with biotinylated detection antibody at room temperature on the shaker. The plate was washed again 3 times. Diluted streptavidin-PE was then added, and the plate was incubated for 30 minutes at room temperature on the shaker. Wells were rinsed 3 times with wash buffer and resuspended with 100 μL of wash buffer. The plate was incubated for 2 minutes before being read on a Luminex L200 instrument, with the lower bound set to 50 beads per sample per cytokine. For values below the quantification limit, half the minimum value was imputed; for values above, double the maximum value was used.

### Statistics

Our primary analysis aimed to estimate the impact of baseline cytokine levels, CD4+ and CD8+ T-cell counts, and CD4/CD8 ratios on the rate of change in log(copies per million cells) of intact provirus, as well as secondary outcomes involving defective provirus and its subtypes. The primary predictors were baseline cytokine concentrations and T-cell counts at the first available sample during viral suppression. The secondary analysis sought to determine the effect of concurrent cytokine levels, T-cell counts, and CD4/CD8 ratios on the rate of change of intact or defective provirus over time. Here, secondary predictors included the time-varying cytokine levels within individuals across different measurements, allowing for longitudinal estimation of change rates. We used mixed effects models for repeated measures.

We sought to adjust the cytokine measurements for the batch used. For this, we used multilevel mixed effects interval regression (Stata *meintreg* command) to model the batch effects for each cytokine after log_10_ transformation; we then created adjusted values on the log_10_ scale by subtracting the estimated batch effect from each value and replacing it with the average of all 5 batch effects.

To model intact and defective provirus dynamics, we employed random-intercept, random-slope linear regression on log_e_(0.5 + copies per million cells), incorporating a covariance term between the 2 normally distributed random effects, following our previous work [6]. The addition of 0.5 facilitates log transformation when no copies are detected, minimally affecting most observations and preserving the interpretation of back-transformed regression coefficients as multiplicative effects. In line with the parent study [6], we addressed nonlinearity by fitting linear spline models with 1 knot, allowing the rate of change to decelerate after 7 years of suppression; additional knots or different placements did not enhance data fit [6]. Our base model thus estimated a decline rate up to 7 years of viral suppression and a subsequent rate, permitting random variation among individuals in initial suppression levels and the rate of change during the first 7 years.

For the primary analysis, we assessed the impact of baseline cytokines on the rate of change over the initial 7 years of suppression using interaction terms for the covariate with suppression duration. Fixed covariates in these models indicate that cytokine levels at the onset of viral suppression affect subsequent intact or defective provirus dynamics. For the secondary analysis involving cytokine measurements over time on the rate of change in intact and defective virus, we employed more complex interaction terms as previously described [6]. The causation direction for time-varying covariates remains unclear; thus, we recommend evaluating the plausibility of such influences and considering reverse or bidirectional causation possibilities. In secondary analyses, we adjusted the main result (association between baseline Gal-9 and intact virus dynamics) for 4 potential confounders identified in the literature—nadir CD4, baseline CD4/CD8 ratio, baseline backbone ART, and duration of HIV RNA suppression—by including interaction terms between each covariate and time in the model. Due to the reduced sample size and exploratory design, we did not adjust the computed *P* values for multiple comparisons to improve interpretability. While this might increase the risk of false positives, this approach is supported for exploratory studies with hypothesis-driven predictor assessments [9, 10].

To analyze intact or defective provirus rates of change and the impact of covariates, we express percentage effects as 100 × (exp[coefficient] − 1). We log_10_-transformed the cytokine values for clearer interpretation of effects. The estimated rate of change represents the annual percentage decay in the geometric mean level of provirus (exponential decay), with the covariate effect described as the difference in the rate of change. The modeled multiplicative effects are additive on the log_e_ scale, meaning that they multiply together. For instance, if the decay rate is 40% per year (multiplicative effect, 0.60) at a certain baseline cytokine level and the effect per 10-fold increase in baseline cytokine level is +30% (multiplicative effect, 1.30), then the decay rate with a 10-fold higher baseline cytokine level would be 0.60 × 1.30 = 0.78, or 22% per year.

We examined residuals’ normality, linearity, and variance homogeneity. The main analysis revealed nonnormal distribution of residuals, typical in complex data sets, prompting cautious interpretation. Linearity and variance homogeneity assessments indicated no significant violations. Biostatistical analyses were conducted with Stata version 17 (StataCorp LP). Spearman’s ρ coefficient was calculated to explore baseline correlations between intact or defective proviruses and cytokines, visualized with the *corrr* library in R.

### Ethics Statement

The study was approved by the University of California San Francisco Institutional Review Board. All individuals provided written informed consent at the time of enrollment in SCOPE.

## RESULTS

### Characteristics of Study Participants

**Figure 1.**
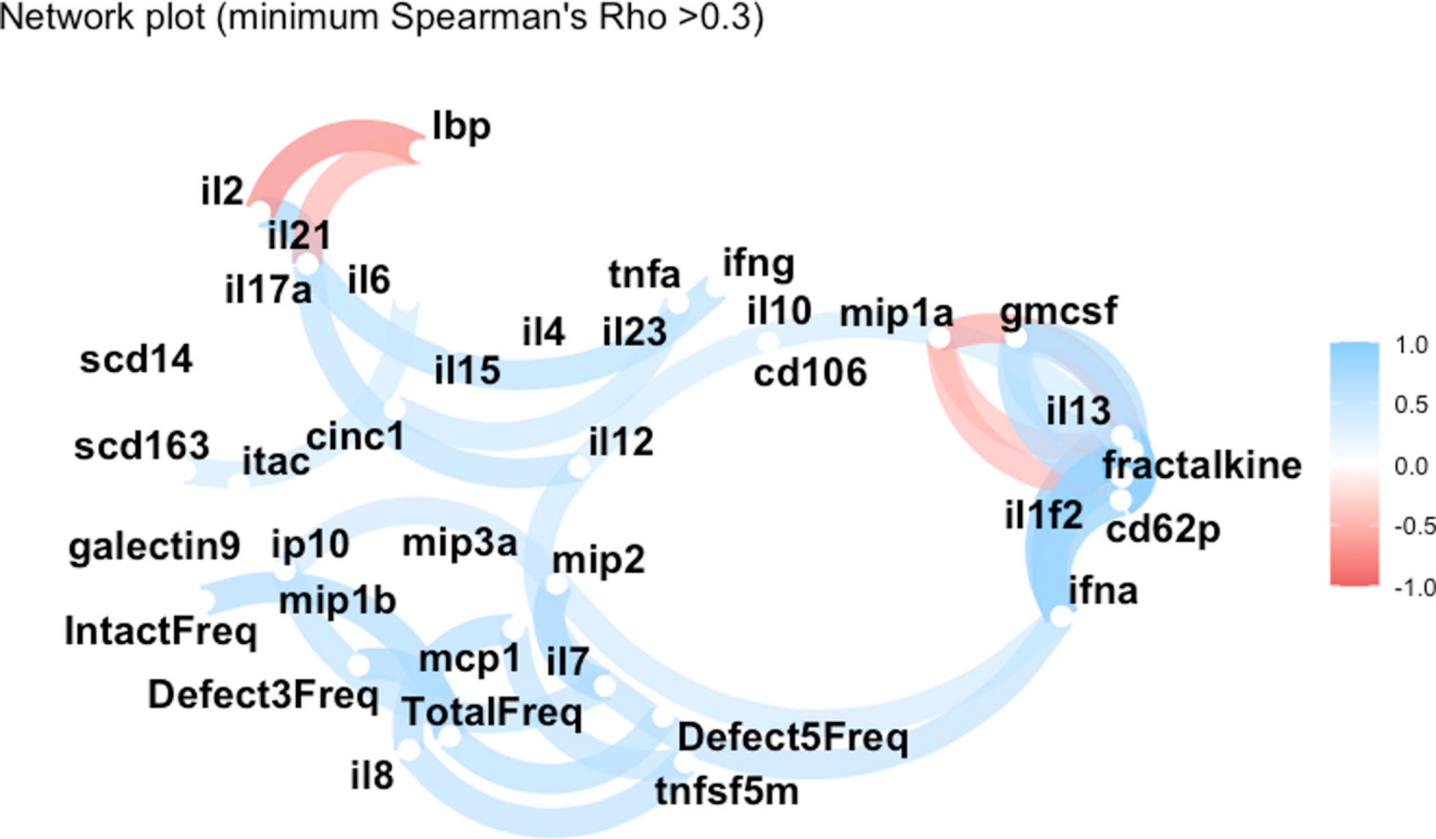
Network plot illustrating baseline correlations (Spearman’s ρ coefficients) between the frequency of intact provirus (IntactFreq) and 3′ (Defect3Freq) and 5′ (Defect5Freq) defective proviruses and baseline cytokine levels. Only those correlations with ρ > 0.3 are represented. Data were obtained from 74 samples, and analysis was run in duplicate.

We analyzed 74 predominantly male participants (94.6%) with a median age of 49 years at the first time point (Table 1). The median nadir and proximal CD4+ T cell counts were 180 cells/μL (IQR, 59–315) and 632 cells/μL (IQR, 459–822), respectively, with a median proximal CD4/CD8 ratio of 0.65 (IQR, 0.44–1.02). The median HIV diagnosis year was 1995 (range, 1981–2011), and the median time from diagnosis to viral suppression was 8 years (IQR, 4.4–12.6). At the time of the first sample analyzed, patients had been undergoing suppressive ART for a median 1.8 years (IQR, 0.3–4.6), with a median follow-up of 7 years (IQR, 5.9–8.3). All patients were prescribed at least 3-drug regimens, with 28 having complex regimens and 48 having standard 3-drug regimens (30 nonnucleoside reverse transcriptase inhibitor based and 18 protease inhibitor based). All maintained ART-mediated plasma HIV RNA suppression during follow-up. Across the cohort, 194 measurements were performed, with an average of 2.6 samples studied per participant. At baseline, intact proviruses correlated with 3′ defective provirus (ρ = 0.61, *P* < .001) and less clearly with 5′ defective provirus (ρ = 0.42, *P* = .079; Figure 1, Supplementary Table 1).

### HIV-1 Provirus Trajectories

**Figure 2.**
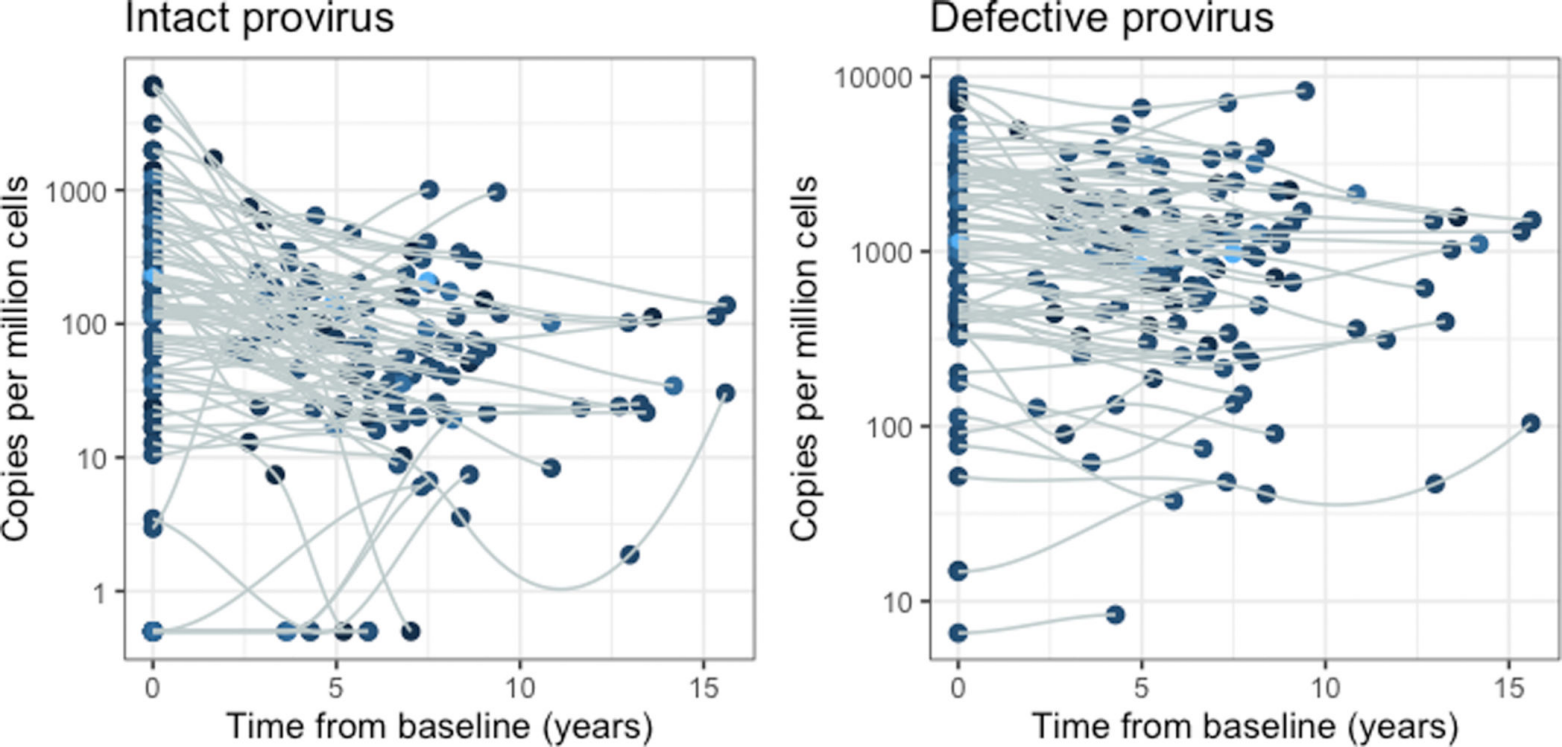
HIV genome trajectories: intact (left panel) and defective (right panel). Data were obtained from 74 samples.

Consistent with previous findings, the intact provirus decayed more rapidly than the defective provirus within the initial 7 years of suppression [6]. Figure 2 illustrates individual and mean trajectories of intact and combined defective HIV proviral DNA in the study participants.

### Effect of Baseline Cytokine Levels on Intact and Defective HIV Decay

**Figure 3.**
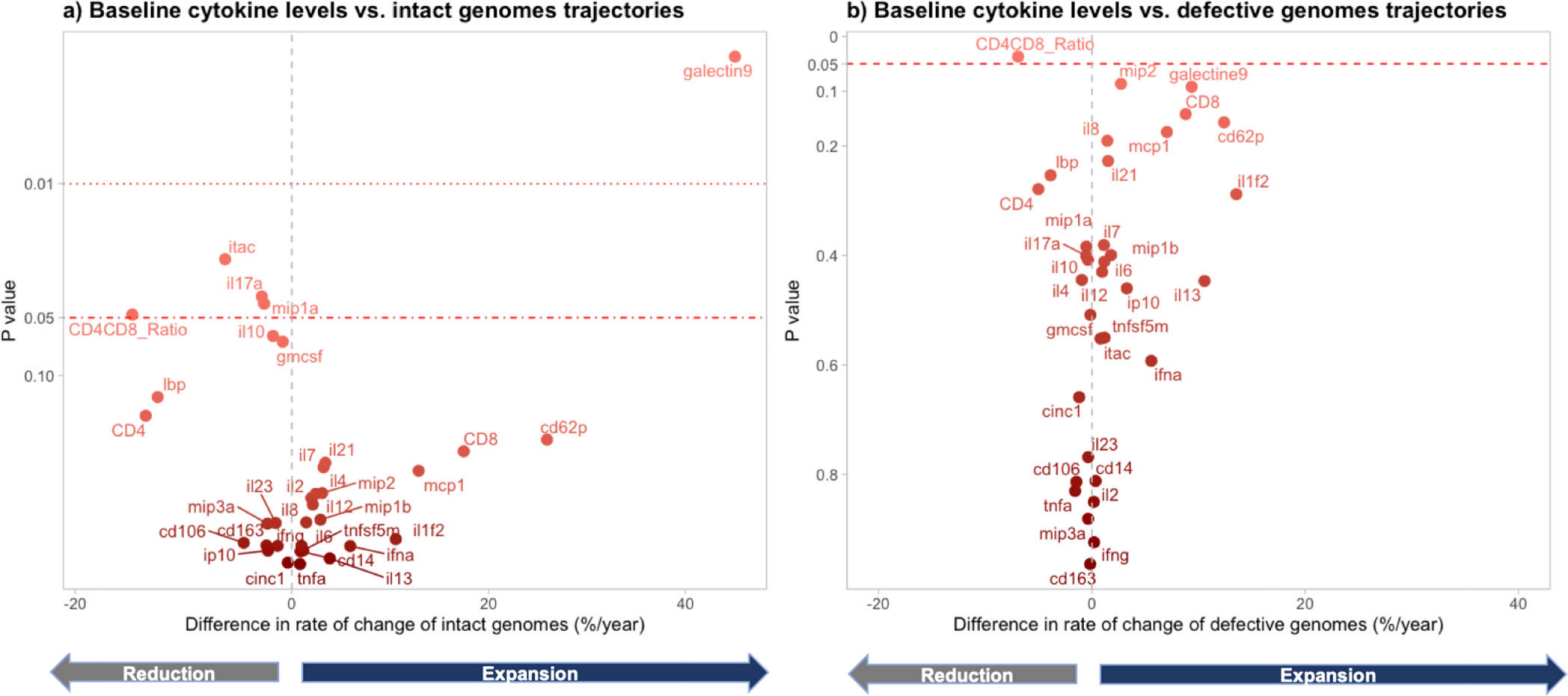
Effect of 10-fold higher baseline cytokine levels on genome trajectories: *A*, intact; *B*, defective. We fitted separate mixed models for each cytokine. The predictors were the log_10_-transformed cytokine concentrations at the time of first available sample during viral suppression. The outcomes were the intact or defective HIV DNA copies per million cells (log_e_ transformed). The x-axis represents the estimated effect on rate of change of intact proviruses per year, after applying the formula 100 × (exp[coefficient] − 1). *A*, For each galectin 9 (Gal-9) 10-fold increase at baseline, there was a mean 45% (95% CI, 14%–84%) greater increase of intact HIV genomes per year. This corresponds to 45% faster decay for each 10-fold lower baseline Gal-9. *B*, For each 10-fold increase of Gal-9 and macrophage inflammatory protein 2 (MIP-2) over time, we observed a mean 9.4% (95% CI, −1.4% to 21.3%) and 2.7% (95% CI, −5.4% to 5.9%) faster increase of defective HIV genomes per year. This corresponds to 9.4% and 2.7% faster decay for each 10-fold lower baseline Gal-9 and MIP-3α. In the opposite direction, for each 10-fold higher CD4/CD8 ratio, there was 6.9% (95% CI, .4–12.9) faster decay of defective genomes per gear. Data were obtained from 74 individuals providing 196 observations. Cytokine measurements were run in duplicate.

In models assessing the impact of baseline cytokine levels on intact HIV kinetics, lower baseline Gal-9 was the strongest predictor of greater subsequent intact HIV decay (Figure 3A, Supplementary Table 2). Each 10-fold decrease in baseline Gal-9 correlated with a mean 45.0% greater decay (95% CI, 14.4%–83.9%) of intact HIV per year. Conversely, increases in baseline interferon-inducible T-cell α chemoattractant (ITAC), IL-17, and MIP-1 predicted slower decay of intact HIV genomes: for each 10-fold cytokine increase, there was a mean decay of 6.7% (95% CI, .9%–12.3%), 3% (95% CI, .2%–5.8%), and 2.8% (95% CI, .1%–5.5%) per year, respectively. Additionally, only the baseline CD4/CD8 ratio predicted intact HIV trajectories among baseline CD4+ T cells, CD8+ T cells, and CD4/CD8 ratio. A 10-fold increase in the CD4/CD8 ratio corresponded to a mean 16.2% greater decay (95% CI, 14.2%–29.7%) in intact HIV. After adjusting for nadir CD4 count, only baseline Gal-9 and ITAC remained significantly associated with intact genome trajectories. Adjusting for potential confounders—including nadir CD4, baseline CD4/CD8 ratio, backbone ART type, and duration of viral suppression—attenuated but did not eliminate the substantial association between baseline Gal-9 and subsequent intact HIV decay: a 10-fold Gal-9 increase was associated with a mean 36.0% greater annual decay (95% CI, 6.3%–72.6%) of intact HIV.

We examined baseline cytokines predictive of defective HIV decay, noting weaker associations (Figure 3B, Supplementary Table 3). Gal-9 and MIP-2 showed the strongest correlations with defective genome trajectories. A 10-fold reduction in baseline Gal-9 levels corresponded to a mean 9.4% (95% CI, −1.4% to 21.3%) and 2.7% (95% CI, 5.4% to 5.9%) greater annual decay of defective HIV, adjusted for CD4 nadir (7.6% [95% CI, 3.6%–17.7%] and 2.8% [95% CI, .2%–5.8%], respectively). Notably, the baseline CD4/CD8 ratio was more strongly associated with changes in defective HIV than soluble cytokines in unadjusted analysis. A 10-fold higher CD4/CD8 ratio resulted in a 6.9% (95% CI, .4–12.9) greater annual decay of defective HIV, adjusted for CD4 nadir (6.8%; 95% CI, .2–5.8).

### Effects of Cytokine Measurements Over Time on Rates of Change in Intact and Defective HIV

**Figure 4.**
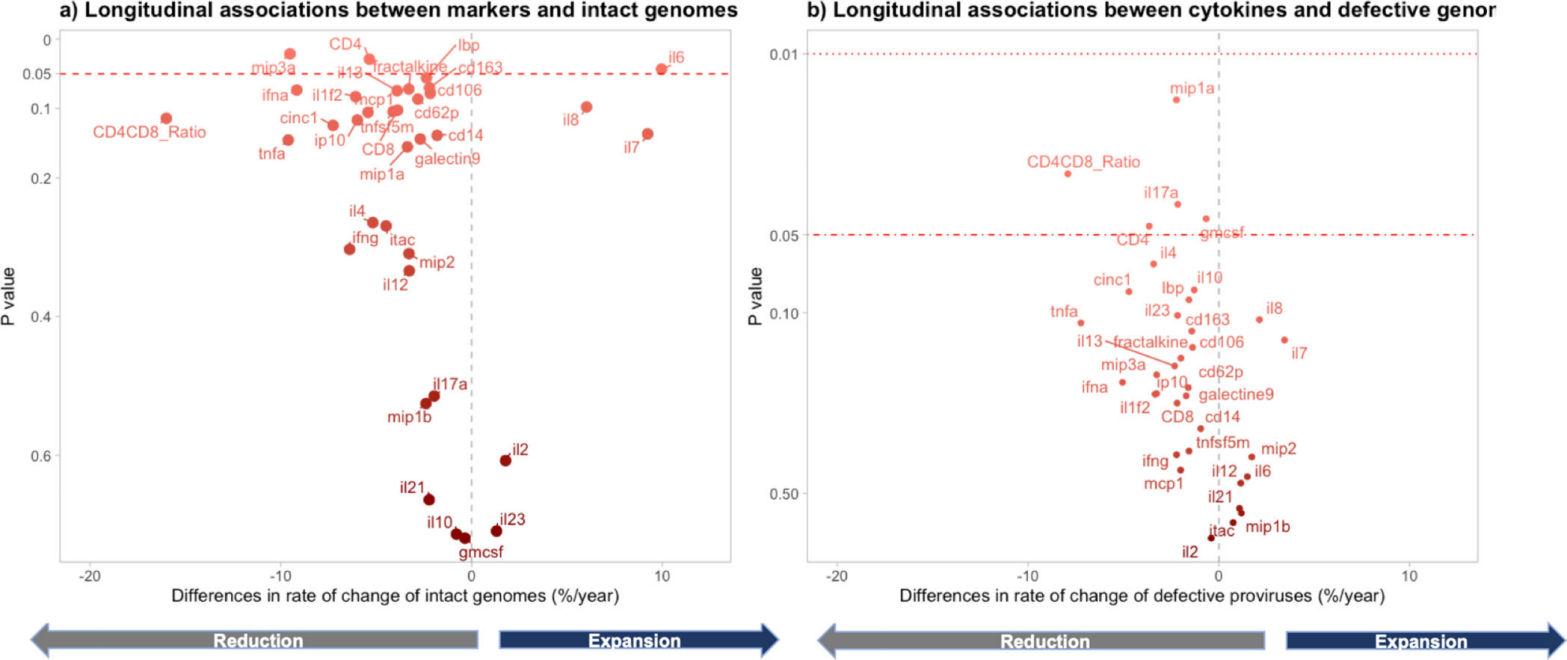
Effects of cytokine changes on genome trajectories: *A*, intact; *B*, defective. We fitted separate mixed models for each cytokine. The predictors were the log_10_-transformed cytokine concentrations longitudinally measured during viral suppression. The outcome was the intact or defective HIV DNA copies per million cells (log_e_ transformed). The x-axis represents the change in rate of change of intact proviruses per year, after applying the formula 100 × (exp[coefficient] − 1). *A*, For each 10-fold increase of interleukin 6 (IL-6) over time, there was a mean 10.0% (95% CI, .3%–20.6%) faster increase of intact HIV genomes. This corresponds to a 10% faster decrease of intact HIV per each 10-fold decrease of IL-6 over time. In the opposite direction, for each 10-fold decrease of macrophage inflammatory protein 3α (MIP-3α) over time, intact HIV decreased 9.5% (95% CI, 1.5%–16.9%) faster per year. *B*, For each 10-fold decrease of the following variables, the mean faster decays of defective HIV were 7.9% (95% CI, .8%–14.5%) for CD4/CD8 ratio, 3.4% (95% CI, .1%–7.1%) for CD4+ T-cell counts, 2.1% (95% CI, .1%–4.1%) for IL-17a, 2.2% (95% CI, .4%–3.9%) for MIP-1a, and 2.2% (95% CI, .4%–4.0) for granulocyte-macrophage colony-stimulating factor. Data were obtained from 74 individuals providing 196 observations. Cytokine measurements were run in duplicate.

MIP-3 and IL-6 had the strongest correlations with longitudinal cytokine changes during ART and intact HIV kinetics (Figure 4A, Supplementary Table 4). A 10-fold IL-6 increase correlated with a 10.0% (95% CI, .3%–20.6%) faster annual decrease in intact HIV. Conversely, a 10-fold MIP-3 increase was associated with a 9.5% (95% CI, 1.5%–16.9%) faster decay of intact proviruses. As previously demonstrated [6], CD4+ T-cell counts were also predictive: a 10-fold increase in CD4 counts over time led to a 5.4% (95% CI, .6%–9.9%) greater decay of intact HIV proviruses.

MIP-1β exhibited the most substantial effect on defective HIV kinetics; each 10-fold increase over time corresponded to a 2% (95% CI, .4%–4.0%) faster decay of defective HIV genomes (Figure 4B, Supplementary Table 5). A cluster including MIP-1α, IL-17, and granulocyte-macrophage colony-stimulating factor also correlated with defective provirus decay, albeit with a weaker effect size. The CD4/CD8 ratio was most strongly associated with defective HIV trajectories, with each 10-fold increase resulting in an 8% (95% CI, .8%–14.5%) greater annual decay of defective HIV.

## DISCUSSION

In this study on the relationship between cytokine levels and the HIV reservoir, baseline Gal-9 was the most predictive marker of intact HIV kinetics over 7 years. A 10-fold decrease in baseline Gal-9 correlated with a 45% greater annual decay of intact HIV genomes, indicating Gal-9’s role in maintaining the intact HIV reservoir and its potential as a therapeutic target to accelerate reservoir decay.

Mechanistically, Gal-9 regulates HIV expression and cytotoxic immunity by modulating cellular stress pathways or activating signaling cascades that enhance HIV transcription from the long terminal repeat. On T cells, Gal-9’s interaction with cell surface oligosaccharides affects transcriptional landscapes, primarily through modulation of key transcription factors, including nuclear factor–κB [11] (central to transcriptional HIV activation) [12], and by activating T-cell receptor downstream ERK and CREB pathways [13]. Studies have shown that Gal-9 is implicated in the reactivation of latent HIV-infected cells in vitro [13, 14], ex vivo [4], and in vivo [15]. Additionally, Gal-9 induces APOBEC3G expression, suggesting a role in antiviral defense by potentially limiting reservoir replenishment when latency is reversed [4], as supported by APOBEC3G’s role in impairing viral replication through hypermutation [16]. Notably, Gal-9’s efficacy in inducing latent HIV-1 transcription may be comparable or superior to other latency-reversing agents [14].

Gal-9 activates and expands several immune cells, including T and myeloid cells), potentially replenishing the HIV reservoir by inducing chronic activation or exhaustion [17]. Previous research in humanized mouse models found that Gal-9 treatment significantly increased tissue-associated HIV DNA and RNA, suggesting that it might expand HIV reservoirs [15]. Our study’s strong correlation between baseline Gal-9 levels and subsequent intact HIV decay implies its detrimental role in HIV persistence, indicating that reducing this protein might accelerate reservoir decay.

Gal-9 may influence host-virus interactions critical for HIV reservoir dynamics, affecting immune checkpoints programmed cell death 1 (PD-1) and T-cell immunoglobulin mucin 3 (TIM-3), which are often upregulated in people with HIV [18, 19]. The Gal-9–TIM-3 axis is significant for modulating T-cell exhaustion, a common feature in chronic viral infections [20]. This regulation could act as a double-edged sword, influencing immune exhaustion markers such as PD-1 in T-cell subsets [21]. The Gal-9–TIM-3 axis may lead to T-cell exhaustion, aiding viral persistence, but it may also set the immune system up for elimination of infected cells [22]. Additionally, Gal-9 modulates the function and proliferation of various immune cells, including CD4+ and CD8+ T cells, natural killer cells, and myeloid cells, with diverse implications for HIV latency. While Gal-9’s effect on CD8+ T cells may boost cytotoxic responses against HIV-infected cells, its influence on CD4+ T cells could affect reservoir size, as these cells are primary targets for HIV infection [14, 21].

In contrast to Gal-9, higher baseline levels of ITAC, IL-17, and MIP-1α correlated with greater decreases in intact HIV, suggesting that these cytokines may promote HIV decay. Further research is needed to elucidate the mechanisms by which these cytokines affect HIV kinetics and their potential as therapeutic targets. ITAC (CXCL11) recruits activated lymphocytes to inflammation sites and is linked to gut epithelial barrier loss and persistent activation during ART suppression [22, 23]. Low ITAC may hinder effective immune cell recruitment to HIV infection sites, contributing to HIV persistence. IL-17, a Th17-cell cytokine, is vital for mucosal immunity and gut barrier integrity, which is compromised in HIV infection [24, 25]. Reduced IL-17 levels might exacerbate gut barrier disruption, enabling HIV translocation and persistence. MIP-1α (CCL3), involved in the inflammatory response and immune cell attraction, has been shown to suppress HIV replication [26]. Like ITAC, lower MIP-1α levels could lead to less effective immune cell recruitment, resulting in higher intact HIV levels [27].

The cytokines measured initially, which predicted later reservoir changes, differed from those that changed simultaneously with reservoir measures. Notably, IL-6 changes were strongly correlated with changes in the intact reservoir. However, the causation direction remains unclear. Elevated IL-6 levels might contribute to HIV reservoir expansion by promoting viral replication through immune activation and cellular proliferation.

CD4/CD8 ratio correlates with HIV reservoir measures, including total HIV DNA, with higher ratios post-ART predicting lower HIV DNA levels [28, 29]. Though less impactful than Gal-9 levels, our findings support utilizing CD4/CD8 ratios to assess immune status in ART-free HIV remission studies. The association between CD4/CD8 ratio and kynurenine/tryptophan ratios suggests IDO-1 activity’s involvement in HIV reservoir decay [30, 31], potentially indicating enhanced mucosal responses or reduced immunoregulatory activity, consistent with decreased Gal-9 levels and improved T-cell and natural killer–cell responses [30].

This study has several limitations. Although this is the largest cohort to date evaluating determinants of long-term changes in the intact reservoir, the sample size remains relatively small. Larger cohorts are needed to confirm our findings. Second, our participants were predominantly men who started ART with a low CD4 nadir. Given the consistent influence of sex [32] and a low CD4 nadir [33], further studies in more diverse populations are essential. Third, we did not adjust the computed *P* values for multiple comparisons, which may increase the risk of false-positive results. However, this approach is supported for exploratory studies [9, 10], where biomarkers (inflammatory cytokines) were specifically selected due to their biological link to our outcome (intact HIV reservoir). Fourth, while we accounted for nadir CD4, CD4/CD8 ratio, type of ART, and duration of viral suppression as potential confounders, the observational nature of our study leaves room for other unaccounted confounders. Future research, particularly experimental studies, would be invaluable in clarifying these relationships and addressing potential confounders. For instance, in vitro latency reactivation experiments in primary CD4+ T cells from samples with varying Gal-9 levels according to established latency reversal assays could elucidate Gal-9’s role in reactivating latent HIV, potentially clarifying its impact on viral transcription and latency clearance dynamics. Our findings on the predictive role of Gal-9 in HIV reservoir kinetics align with previous studies, affirming our results. Although we adjusted for batch effects in cytokine assays, inaccuracies in these adjustments could have affected our results. Batch effects were significant for several cytokines but not for Gal-9. While the analysis of baseline cytokine levels’ impact on long-term HIV DNA changes suggests a causal relationship, the association between these cytokines and HIV persistence does not imply direct influence; other factors linked to cytokine levels might also contribute to HIV persistence.

This study identifies significant differences among various cytokines linked to the evolution of intact and defective HIV reservoirs, underscoring the complex immune responses and diverse dynamics of these reservoirs. The results highlight the unique biology sustaining these 2 subsets and support exploring Gal-9 modulation to target HIV persistence. Additionally, the findings suggest that individuals with lower Gal-9 levels might respond better to strategies aimed at reducing reservoir levels.

## Supplementary Material

Supplementary Tables

## Figures and Tables

**Table 1.** Characteristics of the Study Population

Overall	No. (%) or Median (IQR)
Gender	
Female	3 (3.9)
Male	72 (94.7)
Transgender woman	1 (1.3)
Age at baseline, y^a^	48.8 (9.6)
Race	
African American	9 (11.8)
Asian	1 (1.3)
Latino	6 (7.9)
Mixed	1 (1.3)
Pacific Islander	1 (1.3)
White	57 (75)
Year of HIV infection^b^	1995 (1981–2011)
Years from HIV diagnosis to viral suppression	8 (4.4–12.6)
CD4 nadir, cells/μL	186 (61–320)
Counts at baseline, cells/μL	
CD4	632 (460–815)
CD8	921 (712–1110)
CD4/CD8 ratio at baseline	0.65 (0.46–1.06)
Time from virologic suppression to first sample, y	1.8 (0.3–4.4)
Study follow-up, y	7.0 (5.8–8.3)
Antiretroviral therapy	74 (100)
HIV RNA <40 copies/mL	74 (100)

a Mean (SD).

b Median (range).
